# Mobile phone ownership and willingness to receive mHealth services among patients with diabetes mellitus in South-West, Nigeria

**DOI:** 10.11604/pamj.2020.37.29.25174

**Published:** 2020-09-08

**Authors:** Michael Adeyemi Olamoyegun, Taiwo Hassan Raimi, Oluwabukola Ayodele Ala, Joseph Olusesan Fadare

**Affiliations:** 1 Department of Medicine, Endocrinology, Diabetes and Metabolism Unit, Ladoke Akintola University of Technology, Ladoke Akintola University of Technology Teaching Hospital, Ogbomoso, Oyo State, Nigeria,; 2 Hasso Plattner Institut, Digital Health, University of Potsdam, Potsdam, Germany,; 3 Department of Medicine, Endocrinology and Diabetes Unit, Ekiti State University, Ekiti State University Teaching Hospital, Ado-Ekiti, Ekiti State, Nigeria,; 4 Department of Medicine, Bowen University, Bowen University Teaching Hospital, Ogbomoso, Oyo State, Nigeria,; 5 Department of Pharmacology and Therapeutics, Ekiti State University, Ekiti State University Teaching Hospital, Ado-Ekiti, Ekiti State, Nigeria

**Keywords:** Mobile phone, ownership, diabetes, healthcare, Nigeria

## Abstract

**Introduction:**

mobile phone technology is increasingly used to overcome traditional barriers to limiting access to diabetes care. This study evaluated mobile phone ownership and willingness to receive and pay for mobile phone-based diabetic services among people with diabetes in South-West, Nigeria.

**Methods:**

two hundred and fifty nine patients with diabetes were consecutively recruited from three tertiary health institutions in South-West, Nigeria. Questionnaire was used to evaluate mobile phone ownership, willingness to receive and pay for mobile phone-based diabetic health care services via voice call and text messaging.

**Results:**

97.3% owned a mobile phone, with 38.9% and 61.1% owning smartphone and basic phone respectively. Males were significantly more willing to receive mobile-phone-based health services than females (81.1% vs 68.1%, p=0.025), likewise married compared to unmarried [77.4% vs 57.1%, p=0.036]. Voice calls (41.3%) and text messages (32.4%), were the most preferred modes of receiving diabetes-related health education with social media (3.1%) and email (1.5%) least. Almost three-quarter of participants (72.6%) who owned mobile phone, were willing to receive mobile phone-based diabetes health services. The educational status of patients (adjusted OR [AOR]: 1.7{95% CI: 1.6 to 2.1}), glucometers possession (AOR: 2.0 [95% CI: 1.9 to 2.1) and type of mobile phone owned (AOR: 2.9 [95% CI: 2.8 to 5.0]) were significantly associated with the willingness to receive mobile phone-based diabetic services.

**Conclusion:**

the majority of study participants owned mobile phones and would be willing to receive and pay for diabetes-related healthcare delivery services provided the cost is minimal and affordable.

## Introduction

Diabetes is a major public health problem worldwide with a global burden estimated at 463 million in 2019 and projected to reach 578 million in 2030 and 700 million by 2045 [1]. Over 79% of patients with diabetes globally live in low- and middle-income countries [1]. The prevalence of diabetes in Nigeria is equally increasing at alarming rate both in the rural and urban areas. Various researchers have reported prevalence ranging from 2% to 12% across the country in recent years [2-8]. Diabetes is one of the most expensive chronic diseases in the world due to its life-long nature and complications [1]. These diabetes-related complications arise as a result of poor glycaemic control due to non-adherence to medications and clinic follow-up visits [9]. Documented barriers to adherence include poor communication between the healthcare provider and the patient, poor knowledge of the disease condition and the therapy, complexity of the regimen, fear and experience of side effects, costs of medication and lack of conviction on the need for treatment [10-12]. Countries with advanced health system usually have routine line of communication between patients and their health care providers [13], unlike in developing countries where this interaction often involves travelling long distances on a matter that could be settled by a phone call. Traditional measures of educating patients through face-to-face interactions are becoming challenging due to the excessive healthcare system workload exacerbated by a shortage of manpower. Diabetes self-care management empowers patients with adequate knowledge to participate in their own treatment, using combination of tools like brochures, leaflets and posters. This is currently being augmented with several forms of distance education using various technological measures [13,14].

Mobile phone technology can help reduce the cost of diabetic care among under-privileged people in developing countries as commuting and waiting in hospitals will be reduced significantly. This growing ubiquity of mobile phones holds a promise of using mobile technologies for health interventions. Mobile phones are popularly used by people due to its salient features such as availability, affordability, portability, flexibility, usefulness and simplicity. It has been widely accepted by people irrespective of age, class, socio-economic status and location. It has the potential to empower people by providing efficient and useful information which strengthen, remove physical barriers to care and service delivery while overcoming the health worker shortages [15,16]. Mobile phone enables exchange of information through several means such as text messaging, voice calling, use of social media like WhatsApp, Twitter and emails. It serves as a medium for propagating messages with regards understanding the features, risk factors and complications of diabetes [17]. Mobile phones (especially smartphone) have gained popularity in diabetes prevention and follow-up intervention programs in developed countries, where they are being used to implement actions to promote a healthy diet, regular physical activity and to prevent obesity, enhance the engagement of patients to diabetic care as well as to reduce the number of care drop outs and improve glycaemic control [18-22].

Diabetes is considered as an important medical condition for mHealth interventions, yet it remains under-explored in sub-Saharan Africa [23] especially in Nigeria in the management of diabetes care. There has been a significant growth in mobile phone penetration, since mobile phone services were introduced in Nigeria in 2001 with over 180 million mobile subscribers in the year 2019 which represents a penetration rate of 87% of the population [24]. However, there has been no report of mobile phone-based interventions to support and improve diabetes diagnosis, prevention or treatment. The global pandemic of corona virus disease (COVID-19) in which there was complete lockdown and restriction of movement presents an unprecedented surge in demand for this form of mHealth care from both patients and providers. This study therefore sought to determine the access to mobile phones, attitude to receiving mobile phone-based health care interventions and to determine factors associated with willingness to receive mobile-based healthcare interventions among people living with diabetes in South-West, Nigeria.

## Methods

**Study design and setting:** this was a descriptive cross-sectional study conducted at three (3) tertiary health institutions in South-West region of Nigeria, from April to August 2019. The involved hospitals are LAUTECH Teaching Hospital, Ogbomoso; Bowen University Teaching Hospital (BUTH), Ogbomoso; and Ekiti State University Teaching Hospital (EKSUTH), Ado-Ekiti. Each of these hospitals is manned by at least an endocrinologist who run diabetes clinic at least once a week.

**Study area:** the South-West region is one of the six geopolitical zones of Nigeria, consisting of six states. The region is predominantly a Yoruba-speaking region with all states being homogenous for a common ancestor and weather condition. The region is considered to be the most educationally advanced geopolitical zone in Nigeria.

**Study participants:** the study participants comprised of patients with diabetes who were attending diabetes clinic at the selected health institutions. A convenient sample size of at least 80-90 participants were recruited from each study sites over a period of three (3) months. Participants were selected consecutively if they met recruitment criteria during the study period. Inclusion criteria were aged 18 years or above, diagnosed with diabetes at least in the last 3 months and currently attending follow up clinic for treatment at the selected centres. Participants were excluded from the study if they had mental health conditions, critical illness that could interfere with response to study questions. Patients with diabetes aged below 18 years old and those with gestational diabetes mellitus were also excluded.

**Data collection:** data was collected by the endocrinologist using a specifically designed questionnaire. The questionnaire contains questions assessing sociodemographic characteristics, diabetes-related questions and comprehensive assessment of ownership of mobile phone, type (basic or smartphones), duration of use, ability to send and receive text messages, etc. Participants were also asked of their willingness to subscribe to mobile phone-based diabetic services. Ethical approval was obtained from the ethics and research committee of each centre. Informed verbal consent was obtained from the participants before the commencement of the study.

**Data analysis:** the Statistical Package for Social Sciences (SPSS Inc., Chicago, IL) version 22.0 (IBM Corp., Armonk, N.Y., USA) was used for data analysis. Descriptive statistics were used to summarize the demographic and baseline characteristics. Continuous variables were summarized as numbers of observed values, means, standard deviation and range. Categorical variables were described as frequency and percentages. Associations between characteristics of participants and the desire to receive mobile phone-based health services were analysed using binary and multivariable logistic regression analysis. A p-value <0.05 was considered to be statistically significant.

## Results

There was a total of 259 participants whose questionnaires were analysed in this study. The study participants’ age ranges from 28 to 75 years, with a mean age of 62.1 ± 12.4 years. There were 169 (65.3%) females, 126 (48.6%) had tertiary education, 195 (75.3%) married, 75 (29.0%) retirees, 247 (95.4%) are of Yoruba extraction and 251 (96.9%) had type 2 diabetes. Majority of participants, 109 (42.1%) earned less than 20,000 Naira (≃50 USD @ 1USD= 400 Naira) and only 23 (8.9%) participants earned more than 100,000 Naira (200 USD) per month (Table 1). The treatment of the treatment as shown in Table 1; while 159 (61.4%) were on oral hypoglycaemic agents (OHA) only, 38 (14.7%) on insulin and others were on a combination of both OHA and insulin (Table 1).

**Table 1:** sociodemographic characteristics of study participants and willingness to pay for mobile phone-based diabetic health services (n=259)

Variables	Willingness to pay		Total	P-value
	Yes, n=188	No, n=71	n=259	
Gender male	73(28.2)	17(6.6)	90(34.7)	0.025
Female	115(44.4)	54(20.8)	169(65.3)	
Age groups less than 30	4(1.5)	2(0.8)	6(2.3)	0.061
30-44	13(5.0)	8(3.1)	21(8.1)	
45-54	43(16.6)	6(2,3)	49(18.9)	
55-64	46(17.8)	15(5.8)	61(23.6)	
65 and above	82(31.7)	40(15.4)	122(47.1)	
Address urban	113(43.6)	33(12.7)	146(56.4)	0.117
Semi-urban	40(15.4)	18(6.9)	58(22.4)	
Rural	35(13.5)	20(7.7)	55(21.2)	
Occupation civil servant	38(14.7)	9(3.5)	47(18.1)	0.191
Public servant	12(4.6)	4(1.5)	16(6.2)	
Farmer	9(3.5)	2(0.8)	11(4.2)	
Artisan	22(8.5)	17(6.6)	39(15.1)	
Retiree	54(20.8)	21(8.1)	75(29.0)	
Others	53(20.5)	18(6.9)	71(27.4)	
Tribe Yoruba	178(68.7)	69(26.6)	247(95.4)	0.715
Igbo	7(2.7)	1(0.4)	8(3.1)	
Hausa	1(0.4)	0(0.0)	1(0.4)	
Others	2(0.8)	1(0.4)	3(1.2)	
Religion christianity	164(63.3)	64(24.7)	228(88.0)	0.520
Islam	24(9.3)	7(2.7)	31(12.0)	
Marital status married	151(58.3)	44(17.0)	195(75.3)	0.036
Single	4(1.5)	3(1.2)	7(2.7)	
Divorced	1(0.4%)	1(0.4%)	2(0.8)	
Separated	1(0.4)	0(0.0)	1(0.4)	
Widow	31(12.0)	23(8.9)	54(20.8)	
Level of education none	23(8.9)	12(4.6)	35(13.5)	0.040
Primary	34(13.1)	20(7.7)	54(20.8)	
Secondary	30(11.6)	14(5.4)	44(17.0)	
Tertiary	101(39.0)	25(9.7)	126(48.6)	
Ave monthly income less than 20000	73(28.2)	36(13.9)	109(42.1)	0.280
20000-50000	59(22.8)	21(8.1)	80(30.9)	
51000-100000	37(14.3)	10(3.9)	47(18.1)	
Above 100000	19(7.3)	4(1.5)	23(8.9)	
Type of DM Type 1	8(3.1)	0(0.0)	8(3.1)	0.077
Type 2	180(69.5)	71(27.4)	251(96.9)	
Duration of DM below 5 years	77(29.7)	41(15.8)	118(45.6)	0.049
6-10 years	53(20.5)	20(7.7)	73(28.2)	
11-15 years	33(12.7)	4(1,5)	37(14.3)	
16-20 years	15(5.8)	3(1,2)	18(6.9)	
Above 20 years	10(3.9)	3(1.2)	13(5.0)	
Mode of Treatment OHA	110(42.5)	49(18.9)	159(61.4)	0.166
OHA+ insulin	46(7.8)	16(6.2)	62(23.9)	
Insulin	32(12.4)	6(2.3)	38(14.7)	
Access to mobile Yes	185(71.4)	67(25.9)	252(97.3)	0.074
No	3(1.2)	4(1,5)	7(2.7)	
Phone type: basic(feature) phone	107(42.5)	47(18.6)	154(61.1)	0.181
Smart phone	76(30.2)	22(8.7)	98(38.9)	
Can you read SMS Yes	140(55.6)	47(18.7)	187(74.3)	0.248
No	43(17.1)	22(8.5)	65(25.7)	
Duration of phone use below 5 years	44(17.5)	26(10.4)	70(27.9)	0.075
6-10 years	48(19.0)	21(8.3)	69(27.3)	
11-15 years	39(15.5)	9(3.5)	48(19.1)	
16-20 years	32(12.7)	7(2.7)	39(15.4)	
Above 20 years	20(7.9)	6(2.3)	26(10.3)	

**Mobile phone-related findings among participants:** Table 2 summarises mobile phone possession/ownership. The majority of participants with diabetes, n=252; 97.3%, reported owning a personal mobile phone, with 154 (61.1%) had basic mobile phone (that is mobile phone with no facility for Wi-Fi or internet access) compared to 98 (38.9%) who owned smartphones. There was no difference in the proportion of mobile phone ownership between males and females: (n=88/90, 97.8% vs n=164/169, 97.0%, respectively). Access to smartphone was highest among those with tertiary educational level, younger age group, higher income and lowest duration of diabetes. Among patients with diabetes who owned mobile phone, the majority (n=188, 72.6%) were willing to receive mobile phone-based health services, with proportion being significantly higher in males than females (81.1% vs 68.1%, p=0.025), married compared to single (unmarried), [77.4% vs 57.1%, p=0.036] and level of education, at least secondary level of education compared to none/primary school education (p=0.040). Preferred mode of mobile communication is voice call, 107 (41.3%), text messages, 84 (32.4%), social media, 8 (3.1%) and email, 4 (1.5%).

**Table 2:** comparison of sociodemographic characteristics between male and female participants on access to mobile phone (n=259)

Variables	Access to mobile		Total	P-value
	Male, n=88	Female, n=164	n=252	
Age groups 29 and below	3(1.2)	3(1.2)	6(2.3)	0.569
30-44	5(1.9)	16(6.2)	21(8.1)	
45-54	16(6.2)	32(12.7)	48(18.9)	
55-64	19(7.3)	41(16.2)	60(23.6)	
65 and above	47(18.1)	70(29.0)	117(47.1)	
Address urban	56(21.6)	86(34.7)	142(56.4)	0.143
Semi-urban	21(8.1)	34(14.3)	55(22.4)	
Rural	13(5.0)	42(16.2)	55(21.2)	
Occupation civil servant	18(6.9)	29(11.2)	47(18.1)	0.750
Public servant	6(2.3)	10(3.9)	16(6.2)	
Farmer	3(1.2)	8(3.1)	11(4.2)	
Artisan	11(4.2)	28(10.8)	39(15.1)	
Retiree	30(11.6)	45(17.4)	75(29.0)	
Others	22(8.5)	49(18.9)	71(27.4)	
Marital status married	77(29.7)	118(45.6)	195(75.3)	0.012
Single	4(1.5)	3(1.2)	7(2.7)	
Divorced	0(0.0)	2(0.8)	2(0.8)	
Separated	0(0.0)	1(0.4)	1(0.4)	
Widow	9(3.5)	45(17.4)	54(20.8)	
Level of Education None	6(2.3)	29(11.2)	35(13.5)	0.015
Primary	15(5.8)	39(15.1)	54(20.8)	
Secondary	14(5.4)	30(11.6)	44(17.0)	
Tertiary	55(21.2)	71(27.4)	126(48.6)	
**Average monthly income**				
Less than 20000	29(11.2)	80(30.9)	109(42.1)	0.001
20000-50000	30(11.6)	50(19.3)	80(30.9)	
51000-100000	15(5.8)	32(12.4)	47(18.1)	
Above 100000	16(6.2)	7(2.7)	23(8.9)	
Type of DM Type 1	4(1.5)	4(1.5)	8(3.1)	0.357
Type 2	86(33.2)	165(63.7)	251(96.9)	
Duration of DM below 5 years	40(15.4)	78(30.1)	118(45.6)	0.299
6-10 years	23(8.9)	50(19.3)	73(28.2)	
11-15 years	16(6.2)	21(8.1)	37(14.3)	
16-20 years	4(1.5)	14(5.4)	18(6.9)	
Above 20 years	7(2.7)	6(2.3)	13(5.0)	
**Type of phone owned**				
Ordinary(feature) phone	48(19.0)	103(40.9)	151(59.9)	0.202
Smart phone	40(15.4)	61(23.6)	101(40.1)	
Duration of phone use below 5 years	19(7.3)	53(20.5)	72(27.8)	0.042
6-10 years	20(7.7)	51(19.7)	71(27.4)	
11-15 years	19(7.3)	30(11.6)	49(18.9)	
16-20 years	21(8.1)	19(7.3)	40(15.4)	
Above 20 years	11(4.2)	16(6.2)	27(10.4)	
Preferred mode of mobile communication: voice call	27(10.4)	80(30.9)	107(41.3)	0.105
SMS	36(13.9)	48(18.5)	84(32.4)	
Both	22(8.5)	34(13.1)	56(21.6)	
E-mail	2(0.8)	2(0.8)	4(1.5)	
Social media	3(1.2)	5(1.9)	8(3.1)	
Preferred language native language	24(9.3)	67(25.9)	91(35.1)	0.015
English	38(14.7)	43(16.6)	81(31.3)	
Both	28(10.8)	59(22.8)	87(33.6)	

Participants preferred languages to receive diabetes-related health education from healthcare providers are; native language (35.1%), English (31.3%) and both languages (33.6%). The use of social media was significantly low among our participants: WhatsApp, 69 (27.4%), Facebook, 51 (20.2%), Instagram, 14 (5.6%), Twitter, 9 (3.6%) (Table 3). Most participants, (92.6%) of those who owned mobile phone always have their mobile phone with them, 26.2% do not receive voice calls from unknown phone callers and 50.8% do not share their mobile phone with anybody. The areas of mobile phone-based diabetic health services that participants would want to receive education included: dietary advice, 70.2%, medication use reminder, 62.8%, advice on lifestyles modification, 51.2% and clinic appointment reminder, 50.2% (Table 2). Problems faced by the study participants while using mobile phone are: poor network, 46.4%, visual impairment, 30.6%, irregular electricity needed for phone charge, 25.8% and insufficient fund to buy airtime, 16.3% (Table 2 and Table 4).

**Table 3:** access to social media options/platforms among diabetic study participants

	Whatsapp	Facebook	Twitter	Instagram
**Level of education**				
None	2(2.7)	1(0.4)	0(0.0)	0(0.0)
Primary	6(8.0)	5(9.3)	1(11.1)	1(7.1)
Tertiary	62(82.7)	45(83.3)	8(88.9)	13(92.9)
**Age groups**				
29 and below	3(4.0)	3(5.6)	0(0.0)	0(0.0)
30-44	9(12.0)	8(14.8)	2(22.2)	1(7.1)
45-54	28(37.3)	19(35.2)	6(66.7)	5(35.7)
55-64	18(24.0)	14(25.9)	0(0.0)	4(28.6)
65 and above	17(22.7)	10(18.5)	1(11.1)	4(28.6)
**Average monthly income**				
Less than 20000	19(25.3)	15(27.8)	1(11.1)	3(21.4)
20000-50000	16(21.3)	12(22.2)	1(11.1)	1(7.1)
51000-100000	26(34.7)	16(29.6)	2(22.2)	6(42.9)
Above 100000	14(18.7)	11(20.4)	5(55.6)	4(28.6)
**Gender**				
Male	32(42.7)	25(46.3)	6(66.7)	7(50.0)
Female	43(57.3)	29(53.7)	3(33.3)	7(50.0)

**Table 4:** comparison of basic phone with smartphone use among study participants and areas of desired mobile phone-based diabetic health services

Variables	Type of phone used		Total	P-value
	Basic	Smartphone		
**Level of education**				
None	28(11.1)	5(2.0)	33(13.1)	0.001
Primary	46(18.3)	6(2.4)	52(20.6)	
Secondary	25(9.9)	17(6.7)	42(16.7)	
Tertiary	52(20.6)	73(29.0)	125(49.6)	
**Age groups**				
29 and below	3(1.2)	3(1.2)	6(2.4)	0.001
30-44	9(3.6)	11(4.4)	20(7.9)	
45-54	20(7.9)	28(11.1)	48(19.0)	
55-64	31(12.3)	29(11.5)	60(23.8)	
65 and above	88(34.9)	30(11.9)	118(46.8)	
**Average monthly income**				
Less than 20000	77(30.6)	29(11.5)	106(42.1)	0.001
20000-50000	52(20.6)	25(9.9)	77(30.6)	
51000-100000	17(6.7)	29(11.5)	46(18.3)	
Above 100000	5(2.0)	18(7.1)	23(9.1)	
Gender				
Male	48(19.0)	40(15.9)	88(34.9)	0.202
Female	103(40.9)	61(24.2)	164(65.1)	

**Determinants of willingness to receive mobile phone-based diabetic health service**: results of the bivariate analyses indicated that educational level, average monthly income, duration of diabetes and participants´ age group were associated with willingness to receive mobile phone-based health service interventions among people living diabetes at a p-value of <0.05. The multivariable logistic regression model identified education status (educated; 1.7 [95% CI: 1.6 to 2.1]), type of mobile phone owned (smartphone; 2.9 [95% CI: 2.8 to 5.0]), possession of glucometer (2.0 [95% CI: 1.9 to 2.1]) and average monthly income (>NGN 20,000.00; 1.9 [1.7 to 2.1]) as factors significantly associated with willingness to receive mobile phone-based diabetic health services (Table 5). Educated participants were 1.7 times more likely to be willing to use mobile phone-based health services than uneducated participants. Diabetic patients who possessed glucometer were 2.0 times likely to be willing than those without glucometer. Participants who owned smartphones were 2.9 times more likely to be willing to receive mobile phone-based health services compared to those who possessed basic phone. Also, participants who earned at least 20,000.00 naira/month were 1.9 times more likely to be willing to receive mobile phone-based health service compared to those who earned less.

**Table 5:** bivariate and multivariate analyses of factors with willingness to receive mobile phone-based health services among study participants

Variable	Willingness (Yes)	Willingness (No)	Crude OR (95% CI)	Adjusted OR (95% CI)
Gender male	74	15	1	1
Female	139	24	0.852(0.421 to 1.722)	0.975(0.871 to 1.092)
Educational status: not educated	22	10	1	1
Educated	186	29	1.343(1.148 to 1.798)	1.795(1.625 to 2.010)
Duration of DM: <5 years	78	13	1	1
>5 years	134	21	0.940(0.446 to 1.982)	0.991(0.893 to 1.101)
Own a glucometer? Yes	142	24	1	1
No	56	10	2.057(1.475 to 2.351)	2.008(1.895 to 2.136)
Type of phone: featured phone	120	27	1	1
Smart phone	84	8	2.423(2.183 to4.977)	2.894(2.810 to 4.987)
Monthly income: <#20000	34	9	1	1
>#20000	124	21	1.640 (1.269 to 2.524)	1.925(1.782 to 2.093)

## Discussion

This study, to the best of our knowledge, represents the first attempt to investigate access to mobile phone and willingness to pay for mobile phone-based health services among individuals with diabetes in Nigeria. Most participants (92.6%) in this study had mobile phone but only few had access to internet service, since majority owned basic mobile phones rather than smartphones, hence, mobile phones were mainly used to make/receive calls and also receive/send text messages. In addition, many of the study participants (72.6%) also indicated their intention and willingness to receive and pay for mobile phone-based health services provided the cost was minimal. This proportion of mobile phone ownership is similar to findings by Boyle *et al*. [25] in New Zealand, and Humble *et al*. [26] in UK which reported a significant number of patients with diabetes who had access to mobile phones. However, the result obtained was much higher than 68.5% obtained by Okoro *et al*. [27] in Ilorin, Nigeria in 2010 among individuals with type 2 diabetes and 70.5% observed in similar studies conducted at the University of Gondar Referral Hospital in Ethiopia [28]. The difference in findings may be due to the difference in timing of the study, differences in socioeconomic and availability and cost of telephoning. In our study, participants generally indicated a preference for phone calls and text message and the acceptability for phone calls was significantly associated with levels of education, such that participants with no education or primary school level education were more likely to prefer phone calls compared to text messages. The preference of phone calls could be explained by several factors including the issue of confidentiality.

This finding is significant in patients´ health education as instructions or advice given by phone calls by health care providers based on patient´s symptoms and complaints can lead to possible decreased incidences of hypo-/hyper-glycaemia emergencies. It can also save families the time and finances needed to reach health care facilities which are usually located tens or hundreds of kilometers away. Hence, there is a need for adaptation of types of mobile phone-based health services (text messages or phone calls) to the target diabetic patients according to their literacy level. Tailoring text messages interventions to fit with those specific needs of diabetics is an example of how this adaptation could be implemented. Contrary to the study by Dobson *et al*. [29] who reported that more than 90% of their participants in UK had access to internet, only 33.5% of our participants had access to internet services on their mobile phones, which parallels the prevalence of smartphones. The lower smartphone ownership and internet use can be explained by lack of skills and poor purchasing power among our diabetic patients. Fadare *et al*. [30] reported that more than 70% of patients with diabetes in Nigeria earned less than NGN 50,000 (125USD) per month. Also in this study, patients with younger age group and higher education levels used mobile phone, social media like WhatsApp and internet more often than those with less than secondary school education levels.

These results agreed with those obtained by Jemere *et al*. [28] among diabetics in Ethiopia and by Miller *et al*. [31] among people living with HIV (PLWHIV) in Baltimore, US who found younger patients were more interested in using mobile phone health services. The observed association is probably due to the fact that improved educational status is likely to lead to better diabetes knowledge with a better access to mobile phone and mobile network [31]. In this study, the majority of the participants (70.6%) in our study stated their intention/willingness to pay for mobile phone-based diabetic health services. Many of the participants also stated that using mobile phone for diabetic-based services would be interesting and help them to better manage their diabetes and expressed intention to use if and when available. This agrees with findings in similar study conducted in Ethiopia [28] where 70.2% of their diabetic participants expressed their willingness to pay for mobile phone-based health services. In this study, the most common areas that patients with diabetes required mobile phone-based health care services were clinic appointment reminder, medication reminder, dietary advice and monitoring of blood glucose level. Given the importance of these self-management activities in the overall outcome of diabetes management, these services will be of immense value to improve diabetes-related outcomes. Hence, this intervention has the tendency to improve adherence to medications, SMBG, diet and regular exercise. Direct consultations through the use of mobile phones can help to reduce on the turnaround feedback since some of the consultations do not require physical movement of patients to health centres.

Previously, health care providers on diabetes were not readily able to offer immediate advice to diabetics in case of an emergency, but now, diabetics can get first-hand information from professional health workers without travelling to the health centre making their management easier. Some of the problems identified by the study participants that could affect the effectiveness and functionality of the mobile phone-based diabetic health care services were irregular electricity to charge their mobile phones, poor network connections, inadequate financial power to regularly buy credits on their phones and unwillingness to pick or answer calls from unknown phone numbers. However, as the personal mobile phones were not free of charge, this could potentially impede their use. Alternative strategies not depending on available phones could be to implement toll-free numbers or distribute special SIM-cards for free calls. However, this study has few limitations. First, the study was conducted in tertiary health institutions located within cities where mobile network coverage is close to 100%. Hence, further studies would be needed before scaling it up to rural and remote areas. Notwithstanding, study on ownership of mobile phones in rural communities in Kwara State, Nigeria [32] demonstrated that high proportion (80%) of the dwellers owned phones. Second, a bias could have also been introduced with the cross-sectional nature of the study and the fact that recruitment of participants occurred in diabetes clinic, systematically including those who are naturally inclined to be retained in care and to adhere to treatment.

Further research, such as qualitative studies, are needed to better understand expectations and perceptions of diabetic patients on the contents of text messages or phone calls, especially in terms of confidentiality, privacy and respect of human rights. Studies to explore health care providers´ perception of the introduction of mHealth into care could also be done. The patients with diabetes who are the end users of any digital health tool are key factors for any successful implementation of any mHealth design solution hence, their input will be important for a successful implementation while ensuring privacy. Further qualitative studies to determine the frequency, timing and content of the text message in the design of mHealth interventions may be necessary, as these factors could influence efficacy. As part of our recommendation, individuals with diabetes can be encouraged to acquire mobile phone (preferably smartphone) with SIM card as part of their self-care kit in the same way as glucometer is routinely used for home monitoring of glucose control for those who can afford it.

## Conclusion

The present study establishes baseline data and characteristics hence indicate that majority of our patients owned and have access to mobile phone usage.

### What is known about this topic

- Prevalence of diabetes mellitus is increasing worldwide including the low- and middle-income countries;
- Access to diabetic care is low in developing countries.

### What this study adds

- Mobile phone ownership is high among the study participants;
- Many of the study participants will be willing to pay for mobile phone-based diabetic health services provided the charge is affordable.
